# Expression and Function of Androgen Receptor Coactivator p44/Mep50/WDR77 in Ovarian Cancer

**DOI:** 10.1371/journal.pone.0026250

**Published:** 2011-10-13

**Authors:** Martin Ligr, Ruzeen Rohintan Patwa, Garrett Daniels, Lorraine Pan, Xinyu Wu, Yirong Li, Liantian Tian, Zhenxing Wang, Ruliang Xu, Jingjing Wu, Fan Chen, Jinsong Liu, Jian-Jun Wei, Peng Lee

**Affiliations:** 1 Department of Pathology, New York University School of Medicine, New York, New York, United States of America; 2 Department of Urology, New York University School of Medicine, New York, New York, United States of America; 3 NYU Cancer Institute, New York University School of Medicine, New York, New York, United States of America; 4 New York Harbor Healthcare System, New York, New York, United States of America; 5 Department of Pathology, Northwestern University School of Medicine, Chicago, Illinois, United States of America; 6 Department of Pathology, M. D. Anderson Cancer Center, Houston, Texas, United States of America; 7 Department of Cancer Biology, M. D. Anderson Cancer Center, Houston, Texas, United States of America; University of Chicago, United States of America

## Abstract

Hormones, including estrogen and progesterone, and their receptors play an important role in the development and progression of ovarian carcinoma. Androgen, its receptor and coactivators have also been implicated in these processes. p44/Mep50/WDR77 was identified as a subunit of the methylosome complex and lately characterized as a steroid receptor coactivator that enhances androgen receptor as well as estrogen receptor-mediated transcriptional activity in a ligand-dependent manner. We previously described distinct expression and function of p44 in prostate, testis, and breast cancers. In this report, we examined the expression and function of p44 in ovarian cancer. In contrast to findings in prostate and testicular cancer and similar to breast cancer, p44 shows strong cytoplasmic localization in morphologically normal ovarian surface and fallopian tube epithelia, while nuclear p44 is observed in invasive ovarian carcinoma. We observed that p44 can serve as a coactivator of both androgen receptor (AR) and estrogen receptor (ER) in ovarian cells. Further, overexpression of nuclear-localized p44 stimulates proliferation and invasion in ovarian cancer cells in the presence of estrogen or androgen. These findings strongly suggest that p44 plays a role in mediating the effects of hormones during ovarian tumorigenesis.

## Introduction

Ovarian cancer is the fifth leading cause of death from cancer in women, and the second most deadly gynecologic malignancy in the United States [1]. Epithelial ovarian cancer accounts for about 3% of total cancer cases in women. National Cancer Institute estimated that in 2010, 21,880 women would be diagnosed and 13,850 women would die of cancer of the ovary [2].

Ovarian cancer is a group of heterogeneous diseases and consists of different histological types, which can be readily differentiated by histological evaluation [3]. Current clinical guidelines set forth by World Health Organization distinguish eight histological tumor subtypes: papillary serous carcinoma (PSC), endometrioid carcinoma (EMC), mucinous carcinoma (MUC), clear cell carcinoma (CCC), transitional cell carcinoma (TCC), squamous cell, mixed epithelial, and undifferentiated, with serous carcinoma displaying the most malignant phenotype [4], [5]. Genome-wide global gene analysis further defines distinct expression profiles of different types of ovarian cancer [6]. Different histological types of ovarian cancer seem to be regulated by different pathogenic pathways [7]. Most EMC and PSC present moderate to high levels of ER [8], [9], [10] and AR expression [11], [12].

Steroid hormone receptors, such as ER, progesterone receptor (PR), and AR, are involved in the development of endocrine organ cancers, including ovarian cancer [12], [13], [14]. Estrogens are known to be regulators of growth and differentiation in normal ovaries, as well as in the development of ovarian carcinoma, but the mechanism of this hormonal regulation remains ambiguous. Estrogen acts via two nuclear receptors, estrogen receptor alpha (ERα) and estrogen receptor beta (ERβ) that bind to an estrogen response element (ERE) in the promoter region of target genes, regulating their transcriptional activity [15]. Similarly, AR is also a ligand-activated transcriptional factor. The binding of androgen to the AR results in nuclear localization of the hormone-receptor complex together with coactivators and basal transcriptional machinery. Once in the nucleus it then binds to an androgen response element (ARE), regulating the expression of target genes [16].

AR is a prevalent sex steroid receptor expressed in ovarian cancers. Eighty-four percent of tumors express AR, as opposed to only 74% of tumors expressing ER and 41% expressing PR [17]. There is a higher risk of ovarian cancer in post menopause, at which time androgens are the primary steroids secreted by the ovary [18]. High expression of PR is associated with good prognosis in multivariant analysis for ovarian cancer [19]. However, the results are controversial for the correlation of these three receptors with prognosis and survival rate in patients [12], [20].

p44 is a 44 kDa AR-interacting protein, which has been shown to increase AR transcriptional activity. It contains 342 amino acid residues and four putative WD40 repeats [21]. Furthermore, p44 exists in a methylsome complex with arginine methyl transferase 5 (PMRT5), and is also a subunit of the survival of motor neuron (SMN) complex [22]. Due to phosphorylation of its subunit, the SMN complex is active in the cytoplasm, where it promotes U snRNP assembly [23]. The expression and function of p44 protein have been reported in prostate, testicular, and breast cancers. Interestingly, we observed distinct patterns of expression and function in these reproductive organs [21], [24], [25]. We found distinct intracellular localization of p44 in benign (as nuclear protein) and malignant (as cytoplasmic protein) prostate tissue. Nuclear expression of p44 inhibits prostate cancer growth under the influence of androgen [21]. In contrast, p44 was expressed as a cytoplasmic protein in benign breast epithelia and as a nuclear protein in breast cancer. Nuclear p44 promoted breast cancer cell growth in the presence of estrogen [21], [24]. Our findings indicate that p44 functions as a cofactor influencing the organ-specific tumorigenesis in sex steroid hormone-regulated tumors.

In this study, we examined the expression of p44 in benign and malignant human ovarian cells. We found that p44 was differentially expressed in different types of ovarian cancers. In endometrioid and serous ovarian cancers, p44 was expressed as a nuclear protein. However, p44 was expressed as a cytoplasmic protein in benign ovarian (OSE), fallopian tube (FT), and endometrial (EM) epithelia. Our data also indicated that AR and ER play a role in regulation of the nuclear-cytoplasmic translocation of p44 in ovarian cancer and subsequently cancer cell growth and invasion.

## Results

### Expression and cellular localization of AR coactivator p44 in human ovarian cancer: potential regulation of localization by androgen and estrogen

To determine whether p44 is associated with ovarian cancers, we examined p44 expression in benign ovary, endometrial, and fallopian tube tissues and different histotypes of ovarian carcinomas by immunohistochemistry. To determine the cellular localization of p44, the expression of p44 in cytoplasm and nuclei were semi-quantitatively scored separately (see Materials and Methods).

**Figure 1:**
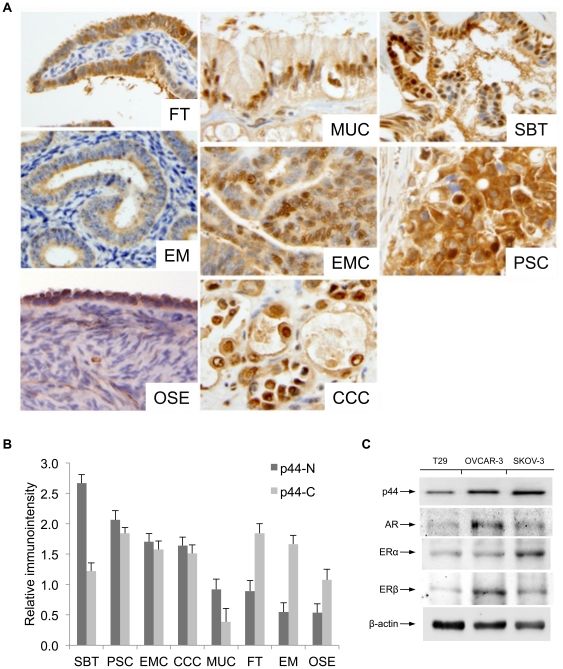
p44 expression and cellular localization in ovarian cancer tissue and matched normal counterparts. A. Examples of p44 expression by immunohistochemistry in 4 different histological types of ovarian cancer and normal fallopian tube and endometrium (magnification 200x). B. Semiquantitative analysis of p44 immunointensity and its cellular localization in four different histological types of ovarian cancer and normal fallopian tube and endometrium. Dark gray bars are nuclear p44 and light gray bars are cytoplasmic p44. Small t-bars represent standard error. C. Western blot analysis of AR, ERα and ERβ expression in different cell lines, including T29, SKOV-3, OVCAR-3. β-actin was used as a loading control. The primary antibody dilution used for p44 was 1∶5000, for AR 1∶1000, for ERα and ERβ 1∶2000 and for β-actin 1∶5000.

p44 was immunoreactive in both cytoplasm and nuclei. In normal tissues, including FT, EM, and OSE, there was higher level of p44 immunoreactive in cytoplasm than in nuclei (cytoplasmic to nuclear ratio was approximately 2∶1, Figure 1B). In all 5 types of ovarian carcinomas, including MUC, CCC, EMC, serous borderline (SBT), and PSC, there was an increase of nuclear immunoreactivity for p44 in comparison to cytoplasm (Figure 1A, Table 1), though nuclear p44 levels varied among different histological types of ovarian cancer. SBT had the highest immunoreactivity for p44 in nuclei and MUC had the lowest (Figure 1B, S1). Multiple ANOVA analysis revealed that there were significant differences of p44 immunoreactivity in nuclei between benign (FT: 0.89±0.17; EM: 0.55±0.15; OSE: 0.54±0.14) and malignant (CCC: 1.64±0.13; EMC: 1.71±0.16; PSC: 2.06±0.14; and SBT: 2.67±0.17) epithelia (p<0.01). Paired t-test revealed that there was significant difference of p44 immunoreactivity in nuclei pair-wise between FT and PSC, OSE and PSC, EM and EMC, respectively (p<0.05). PSC, EMC, and CCC carcinomas showed similar immunointensity of p44 in nuclei by ANOVA analysis (p>0.05, Figure 1B, S1). In contrast, there was minimal difference of cytoplasmic immunoreactivity for p44 between each of benign and malignant epithelia (p>0.05, Figure 1B). In general, EMC, PSC, and SBT had relatively abundant ER expression and these tumors showed relatively higher levels of p44 immunoreactivity in nuclei (Figure 1A, 1B, Figure S1). The findings of difference in cellular localization of p44 between normal tissues and ovarian cancer may suggest a role of p44 as an estrogen receptor mediator in tumorigenesis of ovarian cancer.

**Table 1:** Summary of immunostaining results of p44 and hormone receptors in different types of ovarian cancer and normal control tissues.

	No. of Cases		p44-N	p44-C	ER	PR	AR
**PSC**	32	Mean	2.06	1.84	0.73	0.88	0.21
		Std. Error	0.14	0.14	0.20	0.20	0.11
**EMC**	34	Mean	1.71	1.58	1.92	1.09	0.15
		Std. Error	0.16	0.10	0.24	0.20	0.09
**CCC**	15	Mean	1.64	1.51	1.06	0.52	0.00
		Std. Error	0.13	0.14	0.38	0.25	0.00
**MUC**	15	Mean	0.92	0.38	0.00	0.00	0.00
		Std. Error	0.14	0.14	0.00	0.00	0.00
**SBT**	9	Mean	2.67	1.22	1.59	1.51	0.30
		Std. Error	0.17	0.22	0.48	0.34	0.28
**FT**	30	Mean	0.89	1.84	2.41	2.68	0.00
		Std. Error	0.17	0.16	0.21	0.12	0.00
**EM**	20	Mean	0.55	1.67	1.87	2.28	0.00
		Std. Error	0.15	0.14	0.39	0.36	0.00
**OSE**	28	Mean	0.54	1.08	0.35	0.48	0.25
		Std. Error	0.14	0.18	0.16	0.16	0.12
**Total**	183	Mean	1.55	1.52	1.40	1.23	0.11
		Std. Error	0.08	0.06	0.12	0.11	0.04

**Figure 2:**
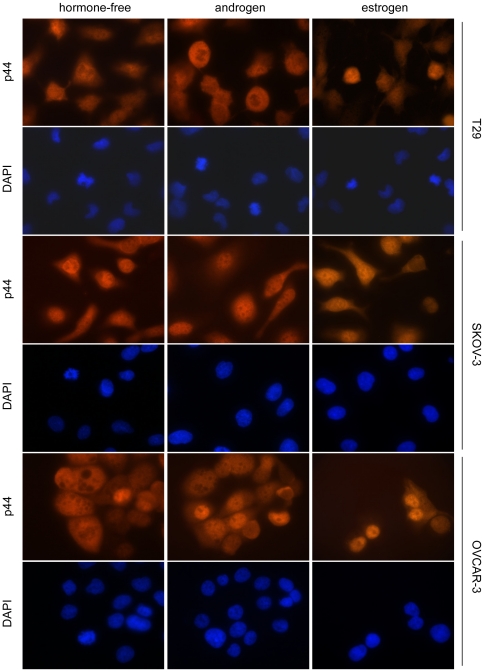
Localization of p44 in T29, SKOV-3, and OVCAR-3 cell lines using immunoflorescence. p44 primary antibody and rhodamine-conjugated rabbit polyclonal secondary antibody was used. DAPI was used to stain the nuclei. (Magnification: 400x)

To evaluate the relationship of p44 with AR and ER in ovarian cancer, we first examined the expression levels of these receptors in the selected ovarian cell lines. Western blot analysis revealed that p44 expression was higher in ovarian cancer cell lines OVCAR-3 and SKOV-3 than the benign ovarian surface epithelial (OSE) cell line T29. The levels of p44 expression positively correlated with ERα/β and AR expression (Figure 1C). In particular, we found that ERα expression was higher in SKOV-3 and conversely, ERβ isoform showed the highest levels in OVCAR-3 cells.To determine p44 cellular localization and regulation by estrogen and androgen in benign and malignant OSE cells, we examined the localization of p44 under three different media conditions: hormone-free medium and medium with defined levels of either androgen or estrogen by immunofluorescence microscopy. As shown in Figure 2, benign T29 cells had higher levels of cytoplasmic p44 and, to certain extent, nuclear p44 in all three media conditions. In SKOV-3, p44 was localized predominantly in the nucleus in hormone-free, androgen (10 nM synthetic androgen R1881) and estrogen (10 nM 17β-estradiol) media (Figure 2). In OVCAR-3 cells, p44 was localized in both the nucleus and cytoplasm in hormone-free media, and located to the nucleus in the presence of either androgen or estrogen (Figure 2).

### AR coactivator p44 functions as both AR and ER coactivator in ovarian cells

**Figure 3:**
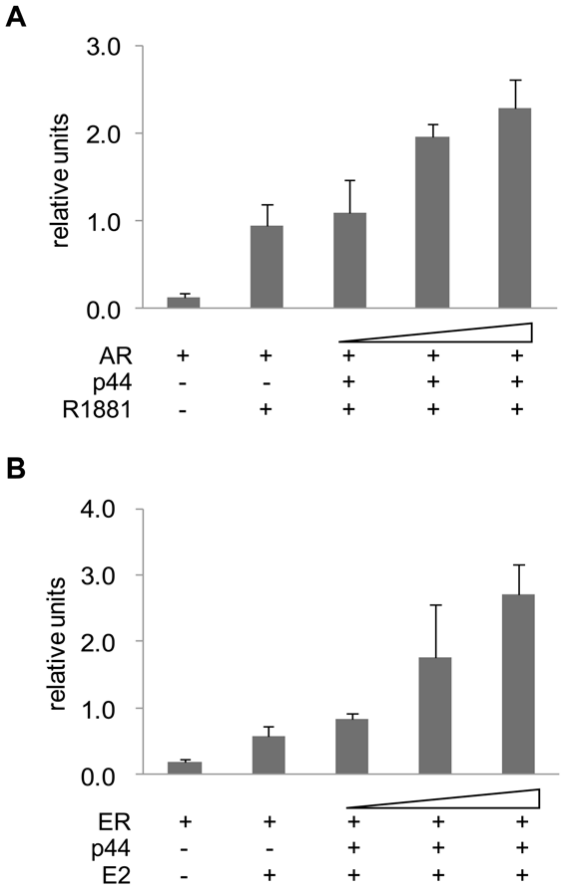
p44 functions as an ERα and AR co-activator in ovarian cell lines. A. Vectors containing p44 and AR were transfected into T29 cells together with a reporter vector containing luciferase gene under the control promoter containing 4 androgen response elements. B. Vectors containing p44 and ERα were transfected into T29 cells together with the ER-luc reporter vector. Reporter activity was expressed as relative units.

p44 is a coactivator of AR and ER and has distinct expression in prostate and breast cell lines [21], [24]. To determine whether p44 functions as a transcription activator in ovarian cancer cell lines, we performed an in vivo transcriptional assay using the dual luciferase reporter system. We transiently transfected T29 cells with vector expressing p44 and either a pair of vectors expressing AR and a luciferase reporter under the control of four androgen response elements (Figure 3A), or ERα, and a luciferase reporter under the control of estrogen response elements (Figure 3B). In the presence of androgen, p44 activated androgen receptor-driven transcription in a dose-dependent manner, leading to as much as 2.6-fold increase of reporter activity compared to the control. Similarly, in the presence of estrogen, p44 activated estrogen receptor-dependent transcription in a dose-dependent manner: the reporter signal increased as much as 4.5-fold compared to control. When p44 was expressed alone, in the absence of either AR or ER, no effect on reporter activity was observed, both in the presence and absence of hormones in the media (data not shown), confirming p44 activation via the hormone receptors. These findings indicated that p44 indeed functions as a coactivator of both androgen and estrogen receptors in ovarian cells.

### AR coactivator p44 promotes ovarian cancer cell growth and invasion

**Figure 4:**
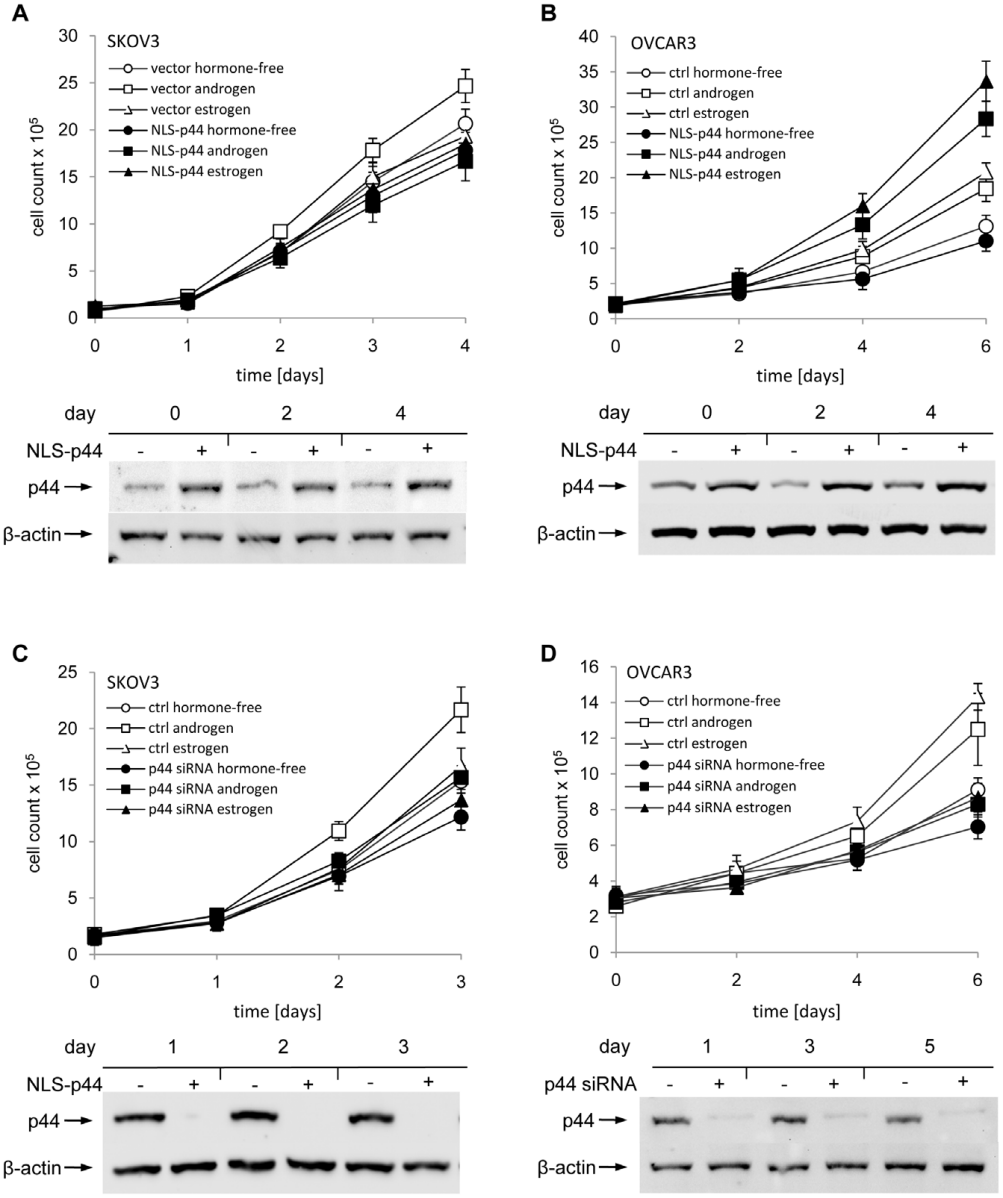
The effect of knockdown and overexpression of p44 on growth of ovarian cancer cells. A,B: SKOV-3 cells (A) and OVCAR-3 cells (B) were transfected either with pBabe-NLSp44 overexpression vector or a control vector, and cultured either in hormone-free medium or in the presence of androgen or estrogen. The overexpression of p44 was verified by western blot (samples taken every other day) and cells were counted every day. C,D: SKOV-3 cells (C) and OVCAR-3 cells (D) were treated with p44 siRNA using Hiperfect and cultured either in hormone-free medium or in the presence of androgen or estrogen. The siRNA treatment was repeated every other day. The overexpression and knockdown of p44 was verified by western blot (samples taken every other day) and cells were counted every day.

To dissect the effect of p44 on ovarian cancer cell proliferation, we first transfected retroviral vectors expressing NLS-p44 into SKOV-3 cells. NLS-p44 is constructed with nuclear localization signal (NLS) fused in frame to N-terminus of p44. SKOV-3 cells are unable to respond to estrogen activation [26], [27]. After confirming that the transfected p44 was expressed, we proceeded to analyze the proliferative status of the cells (Figure 4A). In both hormone-free and estrogen-supplemented media, overexpression of NLS-p44 had no effect on growth of SKOV-3 cells (p>0.05). However, in the presence of androgen, the overexpression of NLS-p44 led to approximately 33% reduction in cell growth (p<0.01). Decreasing the levels of p44 by treating the SKOV-3 cells with the corresponding siRNA led to similar results. While in media containing androgen the knockdown of p44 led to ∼25% decrease of growth rate (p<0.05), and ∼15% reduction of growth of the cells in hormone-free media (p<0.05), there was no statistically significant effect of siRNA treatment on the growth of cells in medium containing estrogen (Figure 4C). Since the estrogen receptor in SKOV-3 cells is non-responsive to estrogen, the results validated our methods of the study.

In contrast to SKOV-3 cell line, the OVCAR-3 cells is responsive to estrogen stimulation [28]. Indeed, when OVCAR-3 cells were grown in media supplemented with estrogen or androgen (Figure 4B and D, open triangles and squares), they both showed increased proliferation compared to cells grown in hormone-free media (55% and 40%, respectively). Contrary to the situation in SKOV-3 cells, we observed a strong positive effect of nuclear p44 overexpression on growth of OVCAR-3 cells in media supplemented wither either androgen or estrogen. In the presence of androgen, the overexpression of NLS-p44 resulted in 1.5-fold increase in growth rate, and in the presence of estrogen the observed growth induction was 1.6-fold. There was no significant influence of cell growth by p44 overexpression in hormone-free media (Figure 4B).

We also observed a strong, but negative, effect of depletion of p44 in OVCAR-3 cells using siRNA confirmed at the protein level by western blot analysis. While overexpression of p44 had no significant influence on growth in hormone-free medium, knock-down of p44 protein levels led to 25% reduction of growth rate in the hormone-free medium. In hormone-supplemented media the growth reduction associated with p44 depletion appeared even stronger. In estrogen media, the p44 depletion caused 40% reduction of tumor cell growth rate, while in the presence of androgen this difference accounted for 33% (Figure 4D).

**Figure 5:**
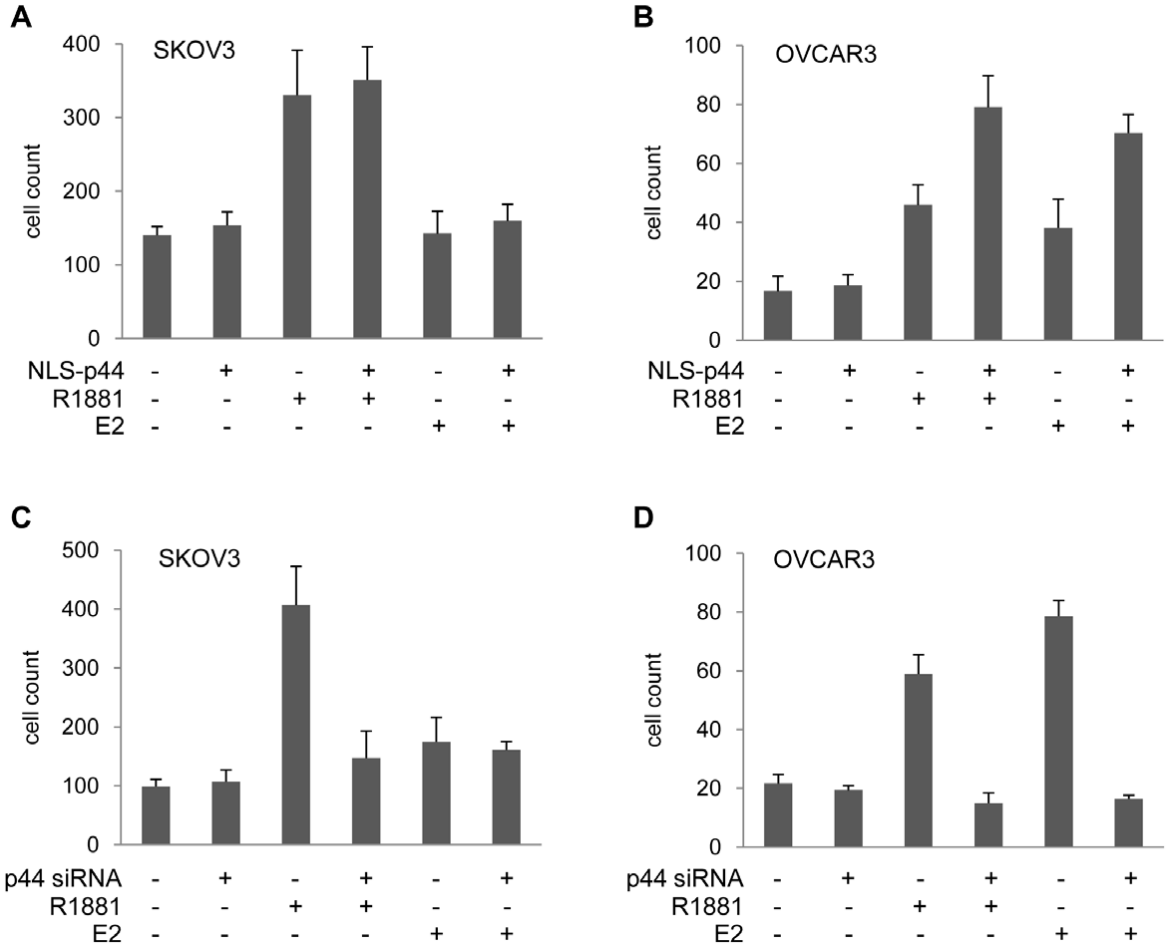
The effect of knockdown and overexpression of p44 on invasive ability of ovarian cancer cells. A,B: SKOV-3 cells (A) and OVCAR-3 cells (B) were transfected either with pBabe-NLSp44 overexpression vector or a control vector; C,D: SKOV-3 cells (C) and OVCAR-3 cells (D) were treated with p44 siRNA using Hiperfect and cultured either in the hormone-free medium or in the presence of androgen or estrogen. The overexpression and knockdown of p44 was verified by western blot (see Figure 4). Cells were seeded onto Matrigel membranes in hormone-free media. Androgen or estrogen were used as chemoatractants in the bottom chamber.

We further tested p44 effects on the cell invasion ability using an *in vitro* Matrigel invasion assay. We observed increased invasiveness of the cells in media supplemented with androgen as compared to hormone-free medium. Treatment of cells with estrogen did not change the number of cells crossing the membrane (Figure 5A). When we overexpressed NLS-p44 in SKOV-3 cells, there was no change in the ability of cells to invade through the Matrigel membrane, either in hormone-free medium or media supplemented with androgen or estrogen. To block endogenous p44 expression, we introduced p44 siRNA into SKOV-3 cells. In media lacking hormones, the depletion of p44 did not affect tumor cell invasion through Matrigel, as it did not affect tumor invasion in the estrogen-containing media. In media supplemented with androgen, lack of p44 expression significantly decreased cell invasion up to 3-fold (Figure 5C).

We also tested the effect of p44 on invasion in OVCAR-3 cells. Similar to SKOV-3 cells, increase or decrease of p44 expression did not affect the cell invasion in hormone-free media (Figure 5B, D). In androgen or estrogen-supplemented media, more than two fold increase of Matrigel invasion was noted (Figure 5B). Consistently, knockdown of p44 in OVCAR-3 cells resulted in a dramatic reduction of invasion ability in the hormone-supplemented media.

## Discussion

Steroid hormones are important factors in the development and progression of ovarian cancer. The hormone receptors and their cofactors are essential for hormone action. Most ovarian carcinomas, including serous and endometrioid types, display varied levels of ER, PR, and AR expression [12], [27]. Expression of sex steroid hormone receptors and their cofactors in ovarian cancer provide the targets for anti-hormone treatments. Thus, it is pivotal to characterize the role of sex hormones and their cofactor-mediated tumorigenesis in ovarian cancer. It has been reported that AR is an important marker in ovarian cancer [29], however, the molecular mechanisms for AR associated aggressive ovarian cancer behavior [30], [31], [32] are still poorly understood.

In our previous studies, we found that AR coactivator p44 plays an important role in both prostate and breast cancers, indicating involvement of the AR pathway in tumorigenesis of these endocrine organs. In this study, we applied similar principles to determine the contribution of p44 to oncogenic functions in ovarian cancer tumorigenesis, in relation to androgen or estrogen. We found that cytoplasmic p44 is highly expressed in benign ovarian, endometrial, and fallopian tube surface epithelial cells. However, in different tumor types, p44 has differential cellular expression patterns, reflected by different levels of cytoplasmic and nuclear localizations. p44 is significantly overexpressed in high-grade serous carcinoma, endometrioid carcinomas, clear cell carcinoma, and serous borderline tumors (Figure 1A). In particular, nuclear p44 expression is significantly higher in ovarian cancer tissues than in their matched benign counterparts, supporting the functional role of nuclear p44 in tumorigenesis of ovarian cancer. Similarly, western blot analysis revealed comparable levels of p44 between OVCAR-3 and SKOV-3 (Figure 1C) when the cells were grown in complete medium (with phenol red and non-charcoal stripped serum), however, in androgen-supplemented medium, immunofluorescence staining revealed that p44 is expressed at highest level in the nucleus (Figure 2). It will be of great interest to determine in future studies whether the levels of ER, AR and p44 expression are associated with specific histological types of ovarian cancer, tumor grade, stages, and survival.

To further define the functional roles of p44, we investigated p44 in benign and malignant OSE cell lines in the presence and absence of androgen or estrogen. We used benign immortalized ovarian surface epithelial cell line T29 and two malignant ovarian cancer cell lines SKOV-3 and OVCAR-3 in this study. It was reported that SKOV3 does not respond to estrogen while OVCAR-3 cells are responsive to estrogen stimulation. In agreement, we found that estrogen did not affect p44 localization, cell proliferation, or invasion in SKOV-3 cells, while estrogen enhanced p44 nuclear localization, cell proliferation, and invasion in OVCAR-3 cells. While both ERα and ERβ are expressed in SKOV-3 cells (Figure 1C), SKOV-3 unresponsiveness to estrogen may be due to mutation in ERα [27], indicating the function of p44 may be mediated by ERα, instead of ERβ.

p44 significantly enhanced malignant OSE cell proliferation and invasion in the presence of androgen or estrogen, indicating that expression of AR or ER are required for p44 to stimulate mitogenesis and invasion (Figure 1C). p44-mediated tumorigenic function seems to be different between prostate and ovarian cancer. In prostate cancer, nuclear p44 inhibits cell proliferation in an androgen-dependent manner. In the ovarian cancer cell line OVCAR-3, nuclear p44 promotes cell growth. In particular, it is noted that nuclear p44 promotes invasion in both androgen and estrogen media. The results from ovarian cancer are similar to what we observed in breast. In breast cancer, nuclear p44 promotes tumor cell proliferation in an estrogen-dependent fashion [24]. Although our siRNA-mediated knockdown experiments demonstrated that our p44 antibody specifically recognized a single protein species in prostate, breast, and ovary, we can’t completely exclude the possibility that highly related but functionally distinct proteins (e.g. splicing variants of p44) act as distinct hormone receptor cofactors in each of these tissues. With this caveat, our findings suggest that p44 may also act through an ER-mediated functional pathway in ovarian tissue.

In summary, our data indicated nuclear p44 is involved in ovarian cancer proliferation and invasion. These processes are regulated by androgen and estrogen. p44 may be a potential target for the treatment of ovarian cancer.

## Materials and Methods

### Case selection, TMA and IHC

The cases were collected after surgery at Northwestern Memorial Hospital and New York University from 1996 to 2009. Approvals of Institutional Research Board (IRB) from both institutions were obtained (exempt). A total of 105 cases of ovarian cancer were collected for this study (Table 1). All PSC and EMC cases were retrieved from the NYU tissue bank and all other types of ovarian cancer, as well as normal control tissues, were retrieved from the NWU tissue bank. This included histological diagnosis of high-grade papillary serous carcinoma (PSC, N = 32), serous borderline tumor (SBT, N = 9), endometrioid ovarian carcinoma (EMC, N = 34), mucinous ovarian carcinoma (MUC, N = 15) and clear cell ovarian carcinoma (CCC, N = 15). Normal control tissues used in this study included: 30 normal fallopian tubes (FT, the closest normal controls for high grade serous carcinoma), 20 normal endometria (EM, the closest normal controls for endometrioid carcinoma), and 28 normal ovaries (OSE, ovarian surface epithelia, as general control tissue).

All carcinomas were ovarian primary. Tissue microarray (TMA) preparation has been described in our previous study [33]. In brief, 0.6 and 1 mm tissue cores were collected from each case of well-preserved tumor sections and high density TMA were prepared in two receipting blocks.

The production, affinity purification, and specificity of the rabbit polyclonal p44 antibody used have been described previously [34]. Preimmune serum was used as control. The specificity of the p44 antibody was confirmed by an siRNA assay that showed reduced p44 expression with p44 RNA interference [21]. The relative levels of p44 expression were scored semi-quantitatively by combination of immunointensity [0 (negative), 1+ (faint), 2+ (weak), 3+ (moderate), and 4+ (strong) expression] and immunopercentage (1 as 0–10%, 2 as 10–50%, 3 as 50–75% and 4 as 75–100%)]. Statistical analyses were performed by t-test.

### Cell culture and cell proliferation assay

The cell lines SKOV-3 [35] and OVCAR-3 [28] were obtained from the American Type Culture Collection. The benign immortalized ovarian surface epithelial cell line T29 [36], as previously described in detail, was maintained in medium 199 and MCDB105 (Sigma). The two ovarian cancer cell lines SKOV-3 and OVCAR-3 were cultured in McCoy's 5a medium (Invitrogen) and RPMI 1640 (Gibco) respectively, supplemented with 10% FBS and 1 U/ml of penicillin and 1 µg/ml streptomycin. To measure the proliferation rate, 2×10^4^ cells were seeded into 6-well plates. At the appropriate time points the cells were treated with 0.25% trypsin and 0.38 mg/ml EDTA (Gibco) and counted using a hemocytometer (Reichert).

### Western blot analysis

Western blot analysis was used to check p44, AR, ERα, and ERβ expression in complete medium, knockdown of p44, and overexpression of p44 plasmids (pBabe vector and pBabeNLSp44) in ovarian cell lines. The cells were either grown in 10 cm dishes, 6 well or 24 well plates. Lysis buffer containing protease cocktail inhibitor (Sigma) in 1∶100 ratio was added to the pellets for resuspension. The protein concentration was measured using Bio-Rad protein assay and appropriate amount of protein was loaded for electrophoresis on the SDS-polyacrylamide gel electrophoresis (SDS-PAGE). The protein was then transferred to nitrocellulose membrane for western blot analysis. The membranes were blocked for 1 hour in 5% nonfat dry milk in Tris buffered saline and Tween 20 (20 mM Tris- HCl, pH 7.6, 150 mM NaCl, and 0.1% Tween 20). Blots were then incubated with antibodies raised against AR, ERα, ERβ (Santa Cruz Biotechnology), p44 and β-actin for 2 hours at room temperature, washed with Tris-buffered saline Tween 20 three times. After the washes, the blots were incubated for 2 hrs with appropriate horseradish peroxidase-conjugated secondary antibody (GE Healthcare). The protein bands were detected by using enhanced chemiluminescence kit (GE Healthcare).

### Immunofluorescent microscopy

T29, SKOV-3, and OVCAR-3 cells were grown at two densities- 3×10^4^ and 1×10^4^ per well for three different media conditions (hormone-free, androgen, and estrogen) on chambered slides. The cells were first rinsed three times in PBS, and fixed for 20 min with 4% paraformaldehyde in PBS at room temperature. After fixation, the cells were permeablized with methanol and acetone (1∶1) and incubated at −20°C for 20 mins. The cells were washed 3 times in PBS and blocked for 1 hour with a solution of 5% BSA in TBS-T. The cells were then incubated with p44 primary antibody (1∶250) for 2 hours at room temperature. Following incubation with primary antibody, the cells were stained with rhodamine-conjugated rabbit polyclonal antibody (1∶500) (Abcam) for 1 hour in the dark at room temperature. For counter staining DAPI was applied (1∶1000) and incubated at room temperature for 5 mins in the dark. The intracellular localization of p44 was then examined using Nikon Digital DXM1200 F microscope with Nikon-ACT program.

### RNA Interference assay

Three siRNAs most effective in reducing the level of p44 protein in the ovarian cells were pooled in equimolar amounts and used in subsequent experiments in general at concentration of 100 nM (Table 2). Scrambled siRNA (Ambion) was used as a control.

Cells were grown in 6 well plates to desired confluency in 2 ml of appropriate medium containing serum without antibiotics. 100 nM of siRNA was diluted in 423 µl Opti-MEM media (Gibco) without serum followed by 75 µl of HiPerfect reagent (Qiagen) and incubated for 10 minutes at room temperature to allow formation of transfection complexes. The cells were then incubated with the transfection complexes under their normal conditions for 48 hours.

**Table 2:** Small interfering RNAs targeting p44.

siRNA number	RNA sequences 5′→3′
#2	CUGUUGAAUUGUGGGAACU[dT][dT]
#6	GAGAGGUAUUCUAGUGGCCUCCGAU[dT][dT]
#8	GGACUCAAGCCUUUCUGAGUUGUUU[dT][dT]

### Electroporation

Three plasmids – pBabe vector, pBabeNLSp44 [21], and GFP (control) – were transfected into cells using electroporation. The cells were grown in three conditions – hormone-free, androgen (10 nM), and estrogen (10 nM). The cells were grown to 80% confluency. The pelleted cells were then resuspended in 100 µl of Nucleofactor solution (82 µl of solution +18 µl of supplement solution) (Lonza) for each reaction. Four ng of plasmid was then combined and cell/DNA suspension was transferred into electroporation cuvette. Nucleofactor program recommended for each cell line by the manufacturer was selected and applied. After the electroporation the cuvette was removed and cells immediately transferred to 10 ml of corresponding medium with FCS and incubated for 48 hrs under normal growth conditions.

### Luciferase assay

In vivo reporter transcription assay was performed using the Dual-reporter Luciferase Assay System (Promega) according to manufacturer's instructions. The light output was measured by Lumat LB9507 luminometer (Berthold). The vectors pcDNA-fAR, pcDNA-ERα, and the reporter vectors ER-luc and pGL3-ARE4 were acquired from Addgene.

### Matrigel invasion assay

Cells were resuspended in the medium without serum at 1×10^5^/ml and 0.5 ml were loaded into the inserts of Biocoat Matrigel 24-well invasion chambers (BD Bioscience). The lower chambers were filled with 750 µl of the medium containing 10% fetal bovine serum as the chemoattractant. After 24 h the inserts were removed and the non-invading cells were cleared away from the upper surface of the membrane using a cotton swab. The cells that migrated to the lower surface of the membrane were stained with Diff Quik stain and counted under a microscope.

### Statistical analysis

The mean values and standard errors were calculated. For two-group comparison, we used Student's t-test and Mann-Whitney U-test. For multiple-group comparison, we used ANOVA analysis. The p values less than 0.05 (p<0.05) were considered statistically significant.

## Supporting Information

Figure S1 **Expression of p44, ER, PR, and AR in ovarian cancer tissue and matched normal tissues.** A: Relative expression (y axis) of ER, PR, AR, and nuclear and cytosolic p44 determined by semiquantitative analysis of immunointensity. The analysis was performed in 4 different histological types of ovarian cancer and normal fallopian tube and endometrium (x axis). Small t-bars represent standard error of measurement. B: Dotplot analysis in 105 ovarian cancer patients. Each dot represents one tumor sample (y axis) with relative expression of the selected gene products, including p44N (nucleus), p44C (cytoplasm), ER, PR, and AR for individual cases in panel A.
(TIF)
