# Correction: Expression and Function of Androgen Receptor Coactivator p44/Mep50/WDR77 in Ovarian Cancer

**DOI:** 10.1371/annotation/fe4fca93-8211-430e-bfe0-8a371b9cc20d

**Published:** 2011-10-31

**Authors:** Martin Ligr, Ruzeen Rohintan Patwa, Garrett Daniels, Lorraine Pan, Xinyu Wu, Yirong Li, Liantian Tian, Zhenxing Wang, Ruliang Xu, Jingjing Wu, Fan Chen, Jinsong Liu, Jian-Jun Wei, Peng Lee

The complete funding information is: "This study was supported by Clinical Translational Science Institute (CTSI)(1UL1RR029893) seed fund to PL, Northwestern Memorial Hospital Dixon Translational Research Fund and Marsha Rivkin Center For Ovarian Cancer Research Award to JJW, and predoctoral fellowships from NYU CTSI TL1 (1UL1RR029893) and NYU Molecular Oncology and Immunology Training grant (T32 CA009161) to GD. The funders had no role in study design, data collection and analysis, decision to publish, or preparation of the manuscript." 

