# Supplementary figures and images for: Expression and Function of Androgen Receptor Coactivator p44/Mep50/WDR77 in Ovarian Cancer
Source: PLoS One. 2011 Oct 13;6(10):e26250. doi: 10.1371/journal.pone.0026250 (PMC3192795; doi:10.1371/journal.pone.0026250)

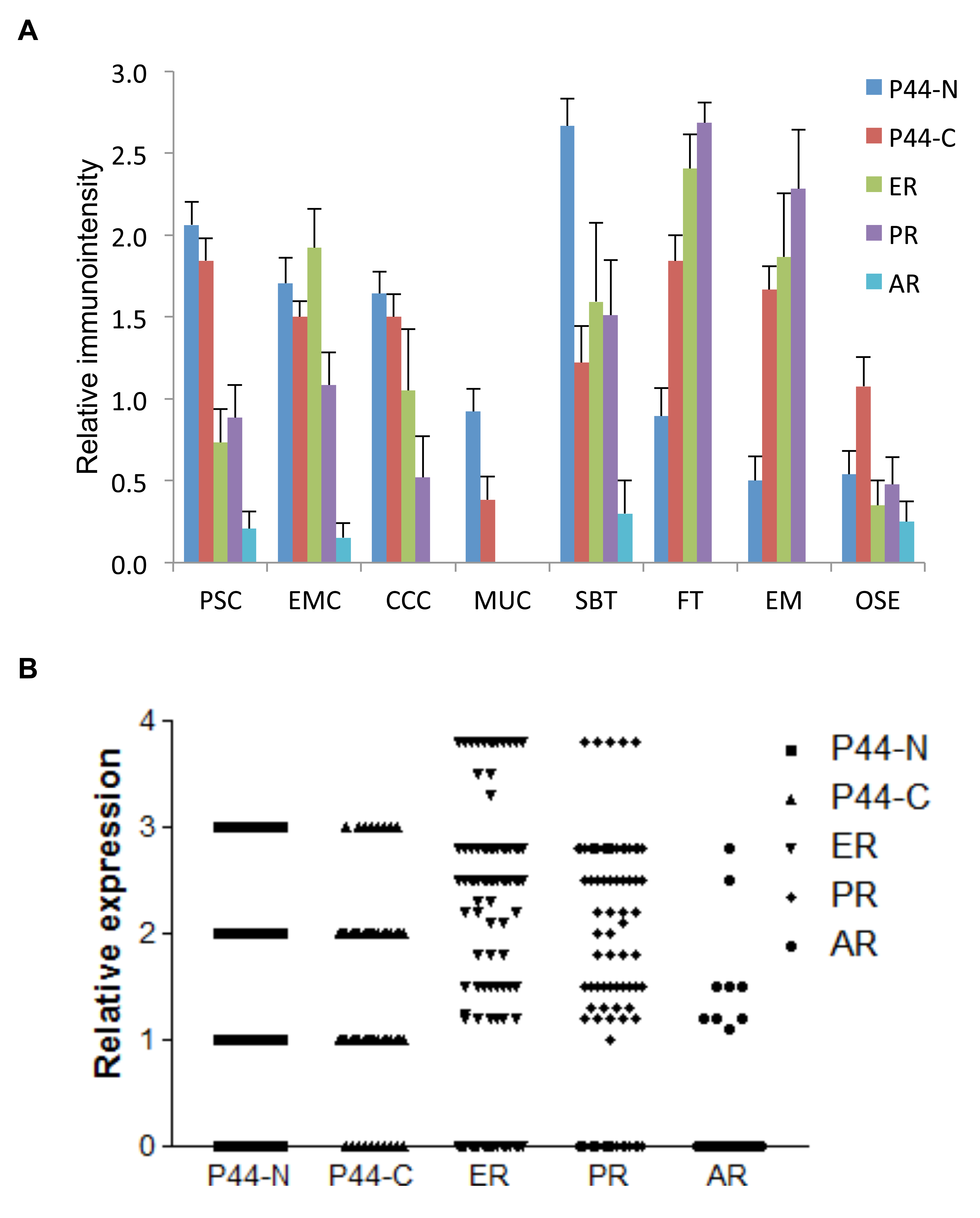

Supplement: Figure S1 — Expression of p44, ER, PR, and AR in ovarian cancer tissue and matched normal tissues. A: Relative expression (y axis) of ER, PR, AR, and nuclear and cytosolic p44 determined by semiquantitative analysis of immunointensity. The analysis was performed in 4 different histological types of ovarian cancer and normal fallopian tube and endometrium (x axis). Small t-bars represent standard error of measurement. B: Dotplot analysis in 105 ovarian cancer patients. Each dot represents one tumor sample (y axis) with relative expression of the selected gene products, including p44N (nucleus), p44C (cytoplasm), ER, PR, and AR for individual cases in panel A. (TIF) [file pone.0026250.s001.tif]
